# A new species of the deep-bodied actinopterygian *Dapedium* from the Middle Jurassic (Aalenian) of southwestern Germany

**DOI:** 10.7717/peerj.5033

**Published:** 2018-06-27

**Authors:** Erin E. Maxwell, Adriana López-Arbarello

**Affiliations:** 1 Staatliches Museum für Naturkunde Stuttgart, Stuttgart, Germany; 2 Department of Earth- and Environmental Sciences, Palaeontology and Geobiology, and GeoBio-Center, Ludwig-Maximilians-Universität München, Munich, Germany

**Keywords:** Middle Jurassic, Actinopterygii, Neopterygii, Dapedium, Opalinuston Formation, Aalenian

## Abstract

*Dapedium* is one of the most abundant and diverse genera of ganoid fishes from the Early Jurassic fossil lagerstätte of Europe. In spite of its abundance, however, its timing of extinction is poorly constrained, with the youngest described material being Early Jurassic in age. We describe new diagnostic and relatively complete material of a large species of *Dapedium* (standard length estimated at 50 cm) from the Middle Jurassic (earliest Aalenian) Opalinuston Formation of Baden-Württemberg, Germany. The Aalenian material represents a distinct species, *D. ballei* sp. nov., differing from Early Jurassic species in a unique combination of characters pertaining to the shape of the dermal skull elements, pectoral fin position, and scale shape and ornamentation. However, although *D. ballei* sp. nov. exhibits a unique combination of characters, there are no autapomorphies with which to distinguish it from the Toarcian species of* Dapedium*. *Dapedium ballei* represents the geologically youngest species of *Dapedium*, extending the range of this genus into the Middle Jurassic. The Opalinuston Formation fills an important gap in the marine vertebrate fossil record, and finds from this horizon have the potential to greatly improve our understanding of evolutionary dynamics over this period of faunal transition.

## Introduction

*Dapedium* Leach, 1822 is a speciose genus of deep-bodied ganoid fish, first appearing in the Late Triassic (late Norian: Tintori, 1983) but reaching its acme in the Early Jurassic, where it is one of the most abundant genera in the Hettangian–Sinemurian Blue Lias and Toarcian-aged Posidonia Shale fossil lagerstätte of England and Germany, respectively (Hauff & Hauff, 1981; Forey, Longbottom & Mulley, 2010). Originally assumed to have a Tethyan distribution (Lehmann, 1966), restudy of material from India now confirms that *Dapedium* is restricted to Europe (Jain, 1973). Fourteen species are provisionally recognised (Table 1), although species named from the Early Jurassic of the UK require re-evaluation and as-yet undescribed species may be present in the Posidonienschiefer Formation (Thies & Waschkewitz, 2016). Species of *Dapedium* were historically classified based on whether the flank scales were smooth or tuberculated, and whether the marginal teeth were bicuspid or unicuspid (Woodward, 1895). However, recently cusp number has been suggested to be relatively variable, although tooth size may represent a useful character for species differentiation (Smithwick, 2015). Thies & Waschkewitz (2016) proposed additional characters for differentiating species from the Toarcian of southwestern Germany, including axial fineness, width of the skull roof, fragmentation or fusion of elements in the orbital series, presence or absence of a presupracleithrum, and gular, opercle, and scale shape.

**Table 1:** Distribution and size of *Dapedium* species currently recognized as valid.

Species	Lithostratigraphy	Age	Length (estimated; mm)	Distribution	Reference
*D. noricum*	Zorzino Limestone	Late Norian	∼80 mm (SL)	Lombardy, Italy	Tintori (1983)
*D. orbis*	“Lower Lias”	Hettangian-Pliensbachian	450 mm	Leicestershire, UK	Woodward (1895)
*D. dorsalis*	“Lower Lias”	Hettangian-Pliensbachian	200 mm	Leicestershire, UK	Woodward (1895)
*D. angulifer*	Wilmcote Limestone Member, Blue Lias Formation	Early Hettangian	∼425 mm (SL)	Warwickshire, UK	Woodward (1895) and Simms (2004)
*D. colei*	Blue Lias Formation	Late Hettangian or early Sinemurian	302 mm (SL)	Dorset, UK	Forey, Longbottom & Mulley (2010)
*D. granulatum*	“Angulatusschichten”; Blue Lias–Charmouth Mudstone Formations	Hettangian–earliest late Sinemurian	∼580 mm (SL)	Dorset, UK; Alsace-Lorraine, France	Woodward (1895) and Forey, Longbottom & Mulley (2010)
*D. politum*	Blue Lias–Charmouth Mudstone Formations	Early–earliest late Sinemurian	325 mm (SL)	Dorset, UK	Forey, Longbottom & Mulley (2010)
*D. punctatum*	Blue Lias–Charmouth Mudstone Formations	Early Sinemurian–?Pliensbachian	344 mm (SL)	Dorset, UK	Forey, Longbottom & Mulley (2010)
*D. radiatum*	Blue Lias–Charmouth Mudstone Formations	Early–earliest late Sinemurian	120 mm (SL)	Dorset, UK	Forey, Longbottom & Mulley (2010)
*D. magnevillei*	“Upper Lias”	Early Toarcian	330 mm	Calvados, France	Woodward (1895) and Wenz (1967)
*D. milloti*		Early Toarcian	290 mm	Yonne, France	Wenz (1967)
*D. pholidotum*	Posidonienschiefer Formation, Schistes de Grandcourt	Early–middle Toarcian	215 mm (SL)	Baden-Württemberg and Lower Saxony, Germany; Calvados, France; Luxembourg	Wenz, 1967, Thies (1988), Delsate (1998) and Thies & Waschkewitz (2016)
*D. caelatum*	Posidonienschiefer Formation	Early–middle Toarcian	400 mm (SL)	Baden-Württemberg, Germany	Thies & Waschkewitz (2016)
*D. stollorum*	Posidonienschiefer Formation	Early–middle Toarcian	350 mm (SL)	Baden-Württemberg and Lower Saxony, Germany	Thies & Hauff (2011) and Klopschar (2017)
*D. ballei* sp. nov.	Opalinuston Formation	Early Aalenian	∼500 mm (SL)	Baden-Württemberg, Germany	This paper

**Notes.**

SL standard length; total length presented if standard length not available

As with many Early Jurassic fishes, the timing of extinction of *Dapedium* is poorly constrained. The relative and absolute abundance of the genus decreases precipitously following the onset of the early Toarcian Oceanic Anoxic Event, and no *Dapedium* remains have been reported from the late Toarcian. Only anecdotal reports of Middle Jurassic (Aalenian) records of *Dapedium* have been cited in the literature (e.g., Schmidt, 1919; Thies & Hauff, 2011), all from the latest Toarcian–earliest Aalenian Opalinuston Formation of Baden-Württemberg, Germany. The fossil record of marine actinopterygians from the earliest Middle Jurassic is particularly poor, with few diagnostic records: aside from fragmentary crania attributed to *Saurorhynchus* (Maxwell, 2016), only isolated teeth (e.g., Delsate & Felten, 2015) and otoliths (e.g., Schwarzhans, 2018) have been reported. Here, we formally describe the actinopterygian remains from the Opalinuston Formation attributed to *Dapedium*, and discuss their significance for faunal change and survivorship of the genus.

### Geological setting

The Opalinuston Formation forms the base of the Middle Jurassic in southern Germany (Dogger *α*), and consists of 100–150 m thick dark, poorly laminated claystones deposited in an epicontinental marine basin. The invertebrate macrofauna comprises bivalves (*Bositra buchi*), abundant gastropods (e.g., *Coelodiscus minutus* and *Toarctocera subpunctata*), ammonites (*Leioceras opalinum*, *Pachylytoceras torulosum*), and brachiopods (*Discina* sp.), with low recorded benthic diversity attributed to soft substrate (Hegele, 1995; Geyer, Nitsch & Simont, 2011; Schulbert & Nützel, 2013). In southwestern Germany, natural outcrops of the Opalinuston Formation are rare, and thus fossil vertebrates from this formation are correspondingly uncommon.

## Materials & Methods

**Figure 1:**
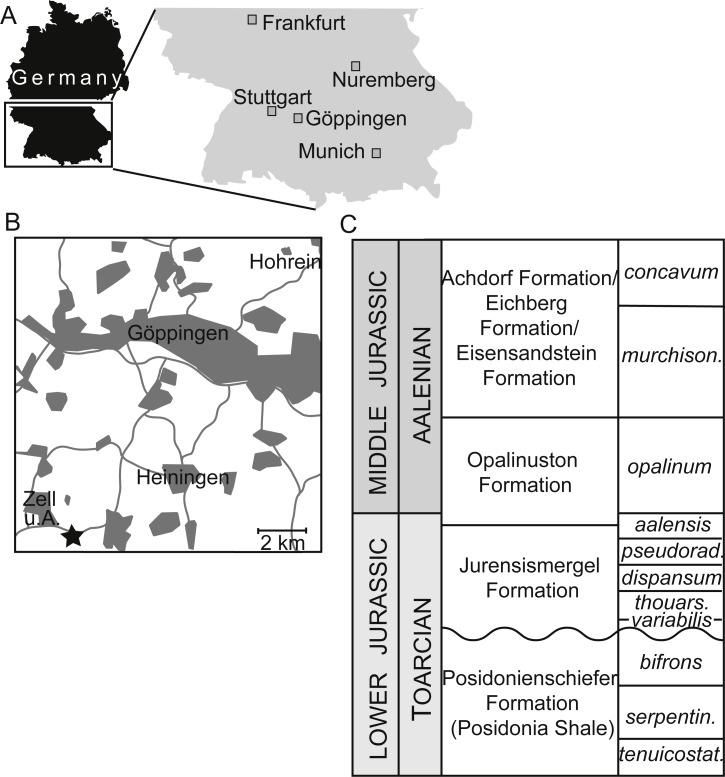
Occurrence information for specimens of *Dapedium* from the Opalinuston Formation. (A) showing the location of the District of Göppingen; and (B) indicating the relative distribution of the localities of Hohrein and Heiningen. The locality of SMNS 96990 near Zell unter Aichelberg is indicated with a star. (C) Biostratigraphic correlation of Toarcian–Aalenian formations in southwestern Germany. Parts (A) and (C) from Maxwell (2016).

Three specimens of deep-bodied ganoid fish have been recovered from the Opalinuston Formation:

1. SMNS 96990. A large fish preserved in a calcareous concretion. The anterior skull is disarticulated; the median fins, pelvic fins, and posteriormost caudal region are absent. The concretion (and fossil) are traversed by multiple cracks infilled with calcite. The concretion is 490 mm long, and was mechanically prepared by Olav Maaß (SMNS). It was collected by Thomas Balle in 2017 from the Opalinuston Formation, which crops out in the Pliensbach (a creek) near Zell unter Aichelberg, Baden-Württemberg, Germany (Figs. 1A–1B). The specimen originates from a horizon approximately 4.5 m above the Jurensismergel-Opalinuston boundary, approximately 1.5 m above the first occurrence of *Leioceras opalinum* at the locality, and so is earliest Aalenian in age (Fig. 1C).
2. SMNS 50167. A partial, fragmentary fish preserved in a calcareous concretion from the Opalinuston Formation, collected in 1974 from a clay pit near Heiningen, Baden-Württemberg, Germany (Fig. 1B). No precise stratigraphic data is available.
3. SMNS 13564. First mentioned by Schmidt (1919), this specimen is the anterior ventral half of a small ganoid fish preserved in a drill core from Hohrein, near Göppingen, Baden-Württemberg, Germany (Figs. 1A–1B). The specimen is preserved in dark shale. Although notes associated with the label suggest that a counterpart was originally present, this has since been lost. The fish originates from approximately 5 m over the Jurensismergel-Opalinuston contact, and is thus probably early Aalenian in age (G Schweigert, pers. comm., 2017). The core log was never published, and the core itself was most likely destroyed during WWII.

In addition, we studied the following specimens first-hand or through high quality photographs (indicated with *). High-resolution original images of specimens housed at the NHMUK have been provided upon request through the Natural History Museum Data Portal (http://data.nhm.ac.uk.); high-resolution images of those holotypes from the British Geological Survey and the Warwickshire Museum were accessed through the GB3D Type Fossils Online project (http://www.3d-fossils.ac.uk).

*Heterostrophus*: *H. latus* SNSB-BSPG AS VI 504; *H. phillipsi* BGS GSM113113*

*Dapedium*: *D. angulifer* WARMS G1120*, *D. caelatum* SMNS 51906, 55869, 56226; *D. colei* NHMUK PV P 1561*, P 4431; *D. granulatum* NHMUK PV P 3538; *D. orbis* NHMUK PV P 4221*, P 29217*; *D. pholidotum* SMNS 51032, 53978, 53989, 54053, 87415, 87405; *D. politum* NHMUK PV P 3555; *D. punctatum*: NHMUK PV OR 36258; *D. radiatum*: NHMUK PV P 1564*; *D. stollorum* SNSB-BSPG 1949 XV 22, SMNS 55858, 56227, 87433.

The electronic version of this article in Portable Document Format (PDF) will represent a published work according to the International Commission on Zoological Nomenclature (ICZN), and hence the new names contained in the electronic version are effectively published under that Code from the electronic edition alone. This published work and the nomenclatural acts it contains have been registered in ZooBank, the online registration system for the ICZN. The ZooBank LSIDs (Life Science Identifiers) can be resolved and the associated information viewed through any standard web browser by appending the LSID to the prefix http://zoobank.org/. The LSID for this publication is: urn:lsid:zoobank.org:pub:2D6609C8-7EFD-4CBD-8ADF-49DB9DA65D97. The online version of this work is archived and available from the following digital repositories: PeerJ, PubMed Central and CLOCKSS.

## Systematic Palaeontology

**Table:** 

Osteichthyes Huxley, 1880
Actinopterygii Cope, 1887
Neopterygii Regan, 1923–24
Dapediiformes Thies & Waschkewitz, 2016

**Table:** 

**Figure 2:**
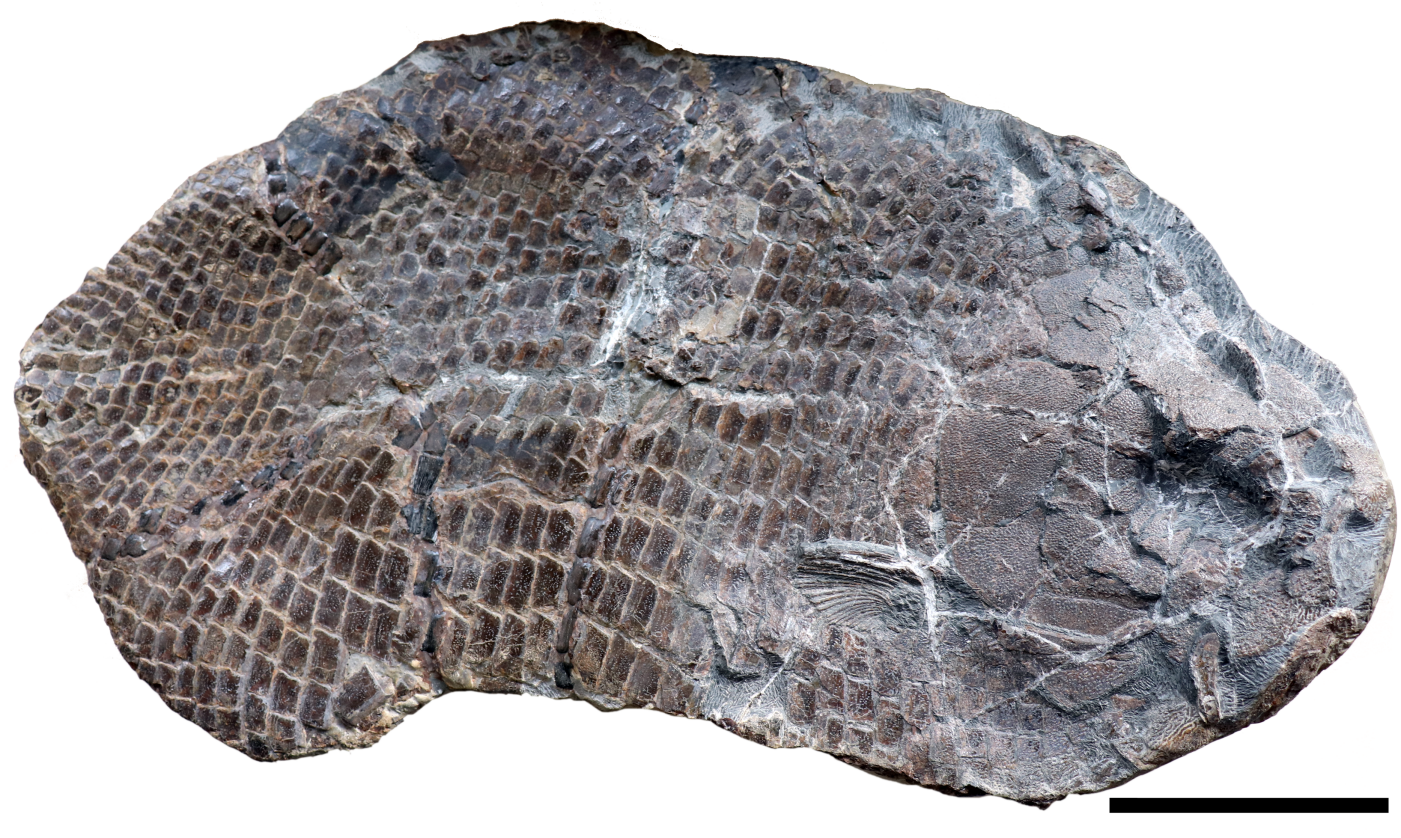
SMNS 96990, holotype of *Dapedium ballei* sp. nov. Scale bar equals 100 mm.

**Figure 3:**
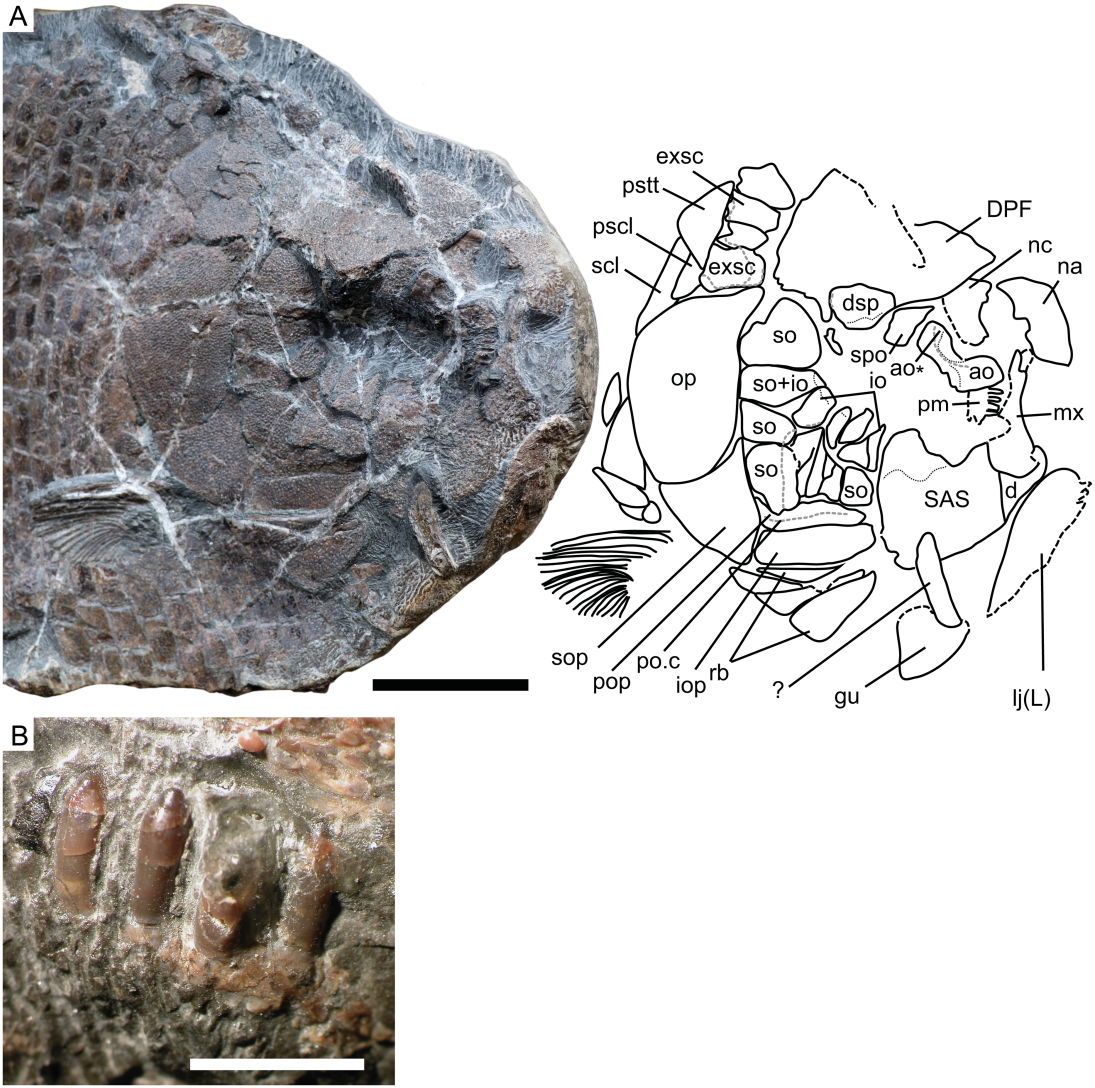
Cranial morphology of *Dapedium ballei* sp. nov. (SMNS 96990). (A) Skull and interpretation, with sensory canals indicated in grey; (B) premaxillary teeth. Scale bar part (A) equals 50 mm, part (B) represents 5 mm. *Abbreviations*. ao, antorbital; ao*, posterodorsal process of the antorbital; d, dentary; DPF, fused dermopterotic-parietal-frontal; dsp, dermosphenotic; exsc, extrascapular; gu, gular; io, infraorbitals, iop, interopercle; lj (L), left lower jaw; mx, maxilla; na, nasal; nc, endochondral neurocranium; op, opercle; pm, premaxilla; pop, preopercle; po.c, preopercular sensory canal; pscl, presupracleithrum; pstt, posttemporal; rb, branchiostegal rays; SAS, fused supraangular-angular-splenial; scl, supracleithrum; so, suborbital; sop, subopercle; spo, supraorbital.

**Figure 4:**
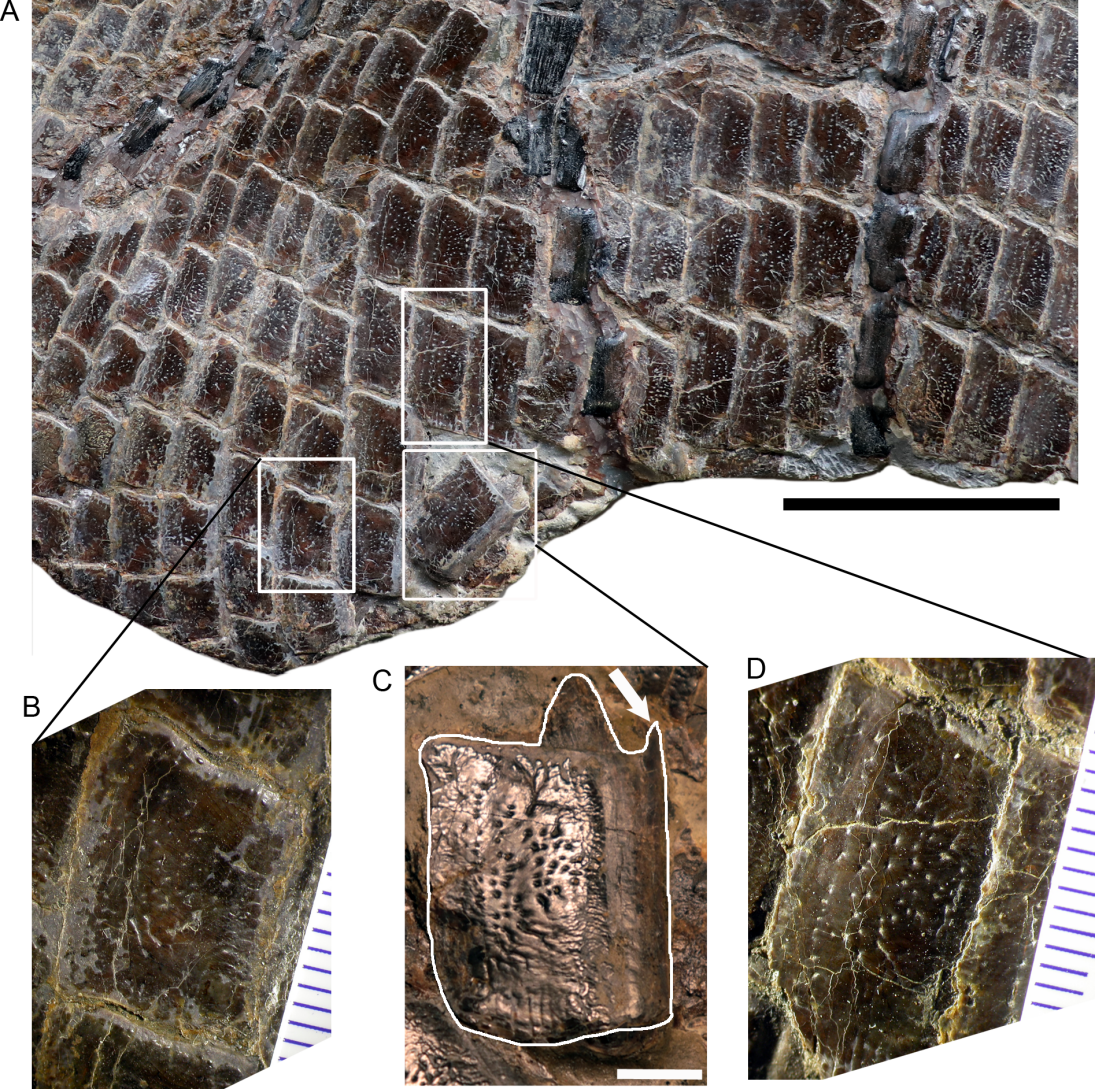
Posteroventral abdominal squamation of *Dapedium ballei* sp. nov. (SMNS 96990). (A) Posteroventral flank, showing dorsoventrally deep scales with punctate ornamentation. (B) Ventrolateral scale showing thinning, patchy ganoine distribution along the posterior margin; (C) disarticulated flank scale illustrating general morphology. The arrow points to the dorsal process protruding from the anterior border of the disarticulated scale. (D) slightly more anterodorsal flank scale than that illustrated in (B) showing thicker ganoine layer ornamented with punctae organised parallel to the posterior edge of the scale. Scale bar (part A) represents 50 mm, that in part (C) represents 5 mm.

**Figure 5:**
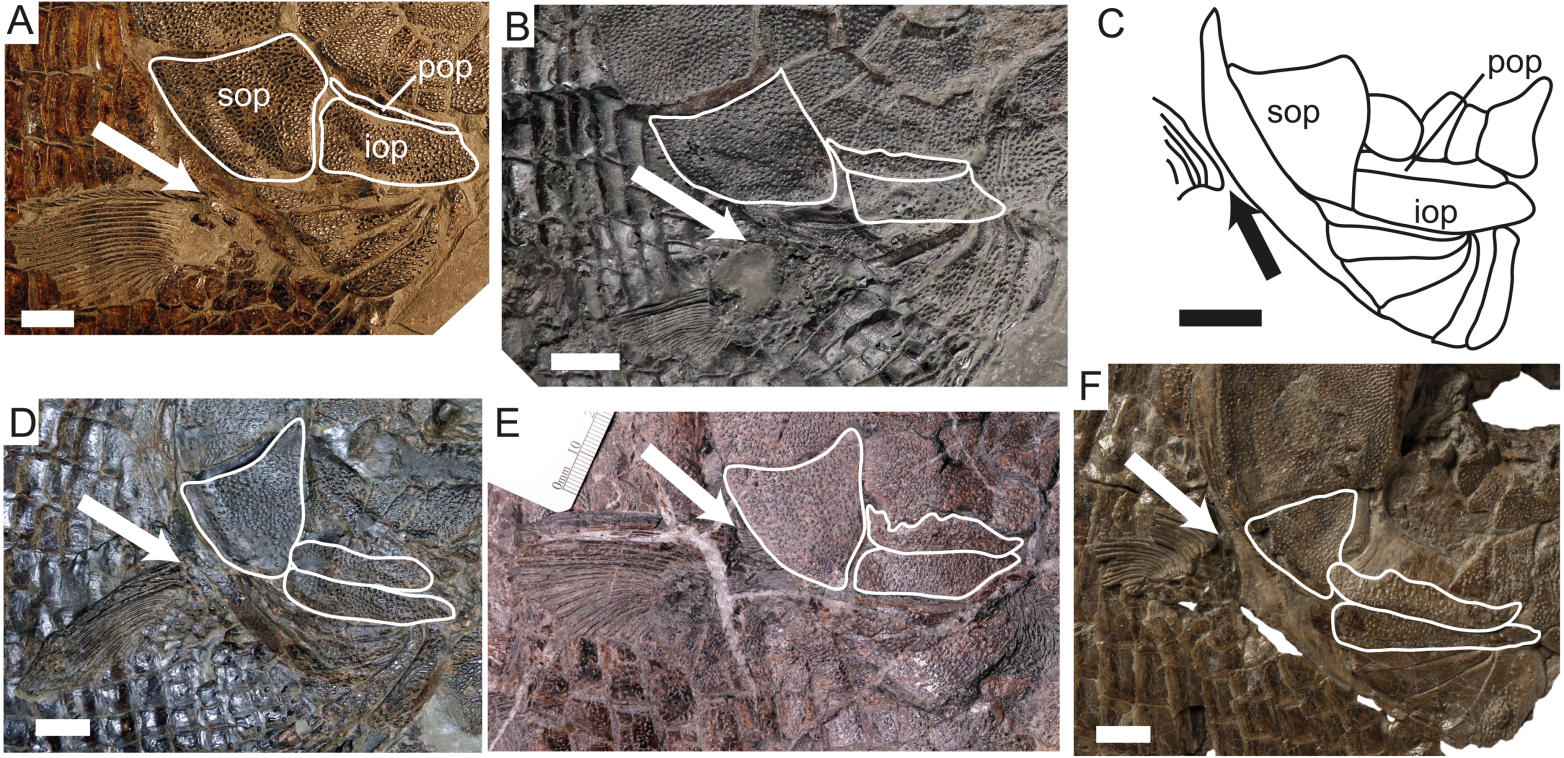
Changes in the opercular series and pectoral fin position through time in dapediid fishes. White outlines indicate the externally exposed portions of the bones bearing tubercular ornamentation. The arrow indicates the dorsal insertion of the pectoral fin. (A) *Dapedium punctatum* (NHMUK PV OR 36258, mirrored); (B) *D. stollorum* (SMNS 87433), note that the pectoral fin is taphonomically ventrally displaced in this specimen and its original position is inferred based on squamation and pectoral girdle morphology. (C) *D. pholidotum*, redrawn from Thies & Waschkewitz (2016) fig. 4; (D) *D. caelatum* (SMNS 51906, mirrored); (E) *D. ballei* sp. nov. (SMNS 96990); (F) *Heterostrophus phillipsi* (BGS GSM113113b). Part (F) modified from http://www.3d-fossils.ac.uk, used under http://creativecommons.org/licenses/by-nc-sa/3.0/. Scale bar represents 10 mm. *Abbreviations*. iop, interopercle; pop, preopercle; sop, subopercle.

**Figure 6:**
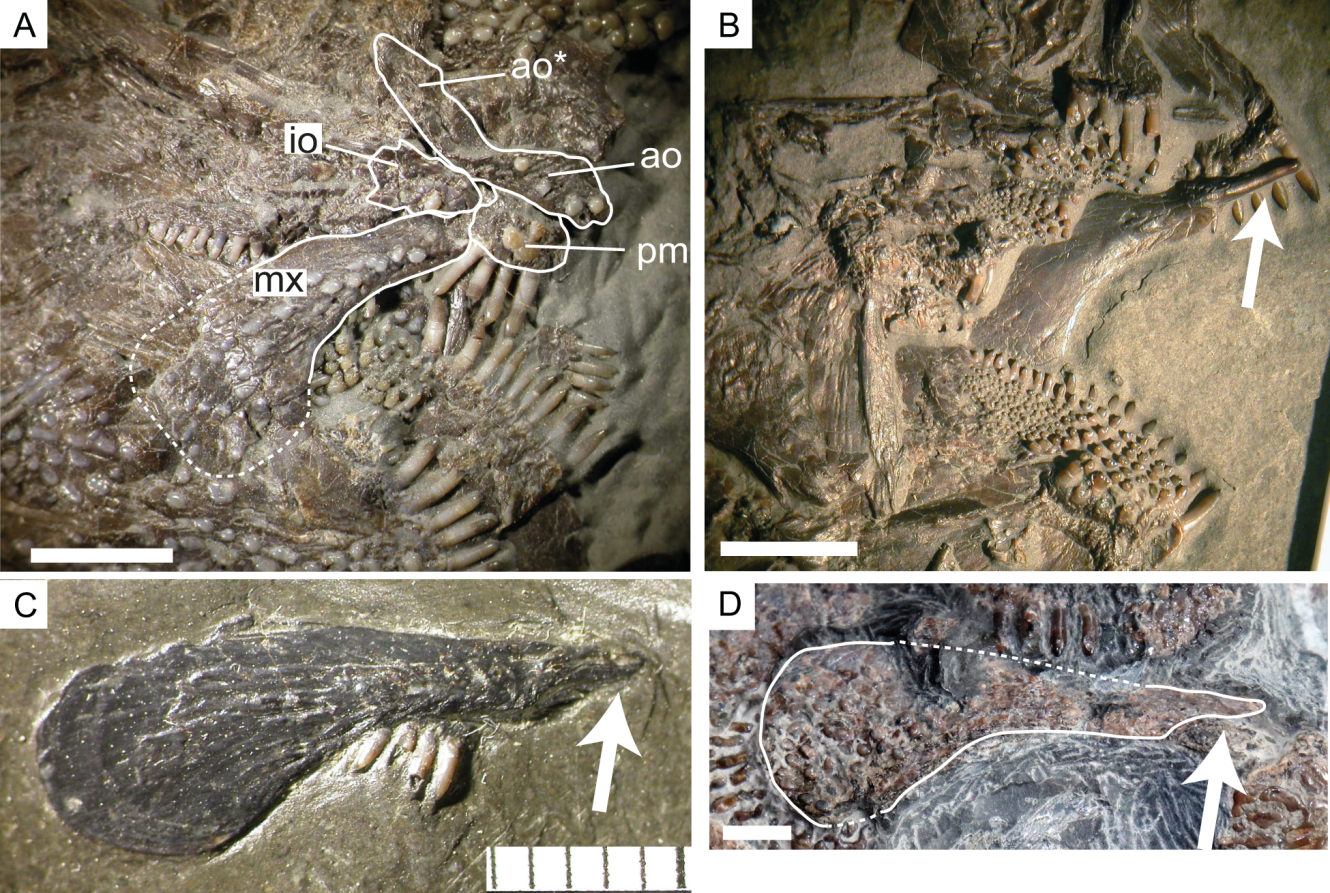
Articular process of the maxilla in *Dapedium*. (A) Fully articulated maxilla in *D. pholidotum* (SMNS 83978); note also the posterodorsal process of the antorbital; (B) maxilla in medial view (*D. pholidotum*, SMNS 87415); (C) a disarticulated maxilla in lateral view (*D. pholidotum*, SMNS 87405); (D) maxilla of *D. ballei* (SMNS 96990) in lateral view. Arrow (parts B–D) indicates the articular process of the maxilla. Scale bar represents 5 mm. *Abbreviations*. ao, antorbital; ao*, posterodorsal process of the antorbital; io, infraorbital; mx, maxilla; pm, premaxilla.

*Dapedium* Leach, 1822
*Dapedium ballei* sp. nov.
urn:lsid:zoobank.org:act:9ADC2848-FD80-4308-AAFE-294C09B0D28D
Figures 2–4, 5E, 6D

**Derivation of name.** Named for Thomas Balle, who collected the holotype specimen and donated it to the SMNS.

**Holotype.** SMNS 96990 (Figs. 2–4, 5E, 6D).

**Diagnosis.** A large species of *Dapedium* distinguished by the following combination of characters: skull bones densely ornamented with ganoine tubercles; dermopterotics, parietals, and frontal bones co-ossified; nasals rectangular, with similarly deep medial and lateral margins; maximal anteroposterior length of nasals c. 0.5 the maximal mediolateral width; bowed antorbital with vertical arm; maxilla with very slender anterior portion; maxillary teeth absent; length of largest premaxillary tooth relative to maximal length of mandible c. 0.07; opercle with a convex ventral contact with the subopercle, and dorsoanterior projection present; depth of the exposed portion of the preopercle about half of the depth of the interopercle; four branchiostegals; medialmost branchiostegal not strongly curved, heavily ornamented with ganoine tubercles, and much wider than lateral branchiostegals; presupracleithrum present; pectoral fin inserting only slightly ventral to the opercle/subopercle suture; trunk scales with pitted surface and smooth posterior border.

### Description

SMNS 96990 has a preserved length of 470 mm, from the anterior mandible to the posteriormost preserved region, interpreted based on scale inflection to lie in the region of the caudal peduncle (Fig. 2). Head length is approximately 148 mm. Assuming a similar head length to standard length ratio as in the Toarcian forms (3.25–3.53: Thies & Waschkewitz, 2016), this gives an SL estimate of 481–522 mm. Body depth is 290 mm, but neither the dorsal nor ventral margins of the fish are preserved. Assuming the SL estimates are reasonable and the depth value is not significantly underestimated, the length:depth ratio is between 1.65–1.8.

**Neurocranium** (Fig. 3). The cranial elements are heavily ornamented with ganoine tubercles, unless otherwise noted.

The extrascapular series includes four elements, as preserved. The lateralmost is the largest. All bear pores related to the cephalic sensory system; in places the canals themselves can be traced. The extrascapulars bear tubercular ornamentation.

The dermopterotic-parietal-frontal (DPF) is cracked in multiple places in SMNS 96990, however no sutures between the elements can be detected. Laterally, ornamentation is tubercular, but tubercles coalesce to form ridges anteriorly and medially. Pores related to the cephalic sensory canals are visible on the DPF around the posterolateral corner of the dermosphenotic. The DPF is 66.9 mm in length; width cannot be accurately measured.

Of the endochondral neurocranium, only the ethmoid region is partially exposed ventral to the DPF, but no details can be described.

A large, convex, roughly rectangular element with a semicircular embayment on one edge is interpreted as the right nasal. The lateral surface bears tubercular ornamentation, but medially the ornamentation becomes coarser, and the tubercles coalesce to form short, irregular ridges. The embayment is interpreted as the nasal contribution to the posterior narial opening. The nasal measures 31 mm along its long axis, and is 15 mm wide at its lateral edge.

**Circumborbital bones.** A curved element preserved anterior to the supraorbital is interpreted as the antorbital. It has been taphonomically rotated and slightly displaced relative to the bones of the dorsal skull roof. The antorbital carries a sensory canal, running the length of the bone and curving dorsally into a short posterodorsal process. The surface of the medial process is ornamented with ganoine tubercles; the lateral surface including the posterodorsal process is unornamented and bears a concavity dorsal to the position of the sensory canal.

The infraorbital series is very difficult to interpret, as it has been disrupted by cracks, sediment displacement, and disarticulation anteriorly. Five narrow infraorbitals are situated along the posteroventral edge of the orbit, with the most posterior element also being the largest. All bear pores related to the cephalic sensory system. Although most infraorbitals are ornamented with tubercles, the posterior two free infraorbitals and the element interpreted as a fused suborbital-infraorbital all have an unornamented portion along their orbital edge showing strong medial curvature into the orbit, and forming the posterolateral orbital wall. A small element posterior to the dermosphenotic and ventral to the descending process of the DPF may also represent an infraorbital, or possibly a dermal component of the sphenotic.

The dermosphenotic is a triangular element along the posterodorsal edge of the orbit. Medially, it contacts the DPF. Its lateral/ventral edge has been damaged. Multiple pores are scattered over the dorsal, posterior, and lateral surfaces of the dermosphenotic, presumably related to the cephalic sensory canals. However, the distribution of these pits does not allow reconstruction of the course of the canals.

The supraorbital is smaller and more elongate than the dermosphenotic, forming the anterodorsal edge of the orbit. It articulates with the anterolateral DPF medially, as well as with the dermosphenotic posteriorly. A few sensory pores are present along its medial edge.

The suborbital series comprises eight plates arranged along the posterior and ventral orbital margins. Based on comparison with other species of *Dapedium*, in which the suborbitals only extend as far anteriorly as the anterior edge of the preopercle, the complete series is likely to be preserved. At least one of these is interpreted as having fused with an infraorbital element. The suborbitals presumably cover the vertical ramus of the preopercle. The smallest suborbitals are situated ventrally.

**Upper jaw.** A dentigerous element that we interpret as a premaxilla is preserved near the antorbital. It is a relatively flat element bearing four tooth positions. Dorsal to the tooth bases, a fossa runs parallel to the dentigerous margin. The element is approximately as long as it is deep.

In lateral view, the maxilla is elongate, with a broad, flat posterior end and narrows anteriorly (Fig. 6D). The ventral edge is concave. The maxilla is edentulous and is ornamented with ganoine tubercles. The maxilla is 39 mm long and 15 mm deep at its deepest point.

**Lower jaw.** Both mandibles are preserved in external view, although the left is heavily weathered and is positioned on the very edge of the concretion. The mandible is 59.8 mm long and 44.1 mm deep at its posterior end. Some impressions of marginal dentition are preserved in association with the left mandible.

The posterior part of the mandible consists of the fused supraangular-angular-splenial (SAS), the individual elements of which cannot be distinguished. The SAS is ornamented with ganoine tubercles, except for the coronoid process, which is unornamented and would have been overlapped by the maxilla. The anterior part of the mandible is made up of the dentary. The suture between it and the posterior SAS is demarcated by a change in ornamentation, with the dentary bearing rugae running parallel to the suture and the SAS ornamented with tubercles. The dorsal portion of the dentary-SAS contact, as well as any teeth, are covered by the anteriorly displaced maxilla.

**Dentition** (Fig. 3B). The only teeth preserved are those on the premaxilla. These are conical, with tall bases and relatively small lingually curving acrodin caps, and appear to be unicuspid. Both the cap and the base are smooth. The best-preserved tooth is approximately 4 mm in height, with a width of approximately 1 mm at the base of the cap.

**Preopercle, opercular bones, branchiostegals and gular.** The preopercle is anteroposteriorly elongate, and carries a sensory canal. Dorsally, it is overlapped by five suborbitals. Posteriorly, it curves slightly dorsally. The laterally exposed portion is ornamented with ganoine tubercles (Fig. 5E). The opercle is deeper than it is long (62 mm high, 43 mm long) and has a sinusoidal anterior-ventral edge and a gently convex posterior border. Anteriorly, it contacts three suborbitals. The subopercle is weakly triangular, with a concave dorsal border that articulates with the convex border of the opercle. Anteriorly, it contacts a single suborbital as well as the pre- and interopercles. The interopercle is anteroposteriorly elongate, tapering slightly anteriorly. It is dorsoventrally deeper than the laterally exposed portion of the preopercle (Fig. 5E).

Four branchiostegal rays are present, the medial-most of which is the largest. Although portions of these bones are lost or overlapping, the borders are clearly well preserved, so the expansion of the medial-most ray is real and not an illusion due to preservation. The externally exposed portions of all rays are heavily ornamented with ganoine tubercles. The medial-most ray is more or less triangular in shape with a slightly convex medial edge which is shorter than the lateral edge. The posterior and lateral edges are essentially straight, showing very little curvature. The more posterior rays are also roughly triangular, except for the lateralmost which is very narrow and shows strong posterior concavity.

The gular plate is preserved at the very edge of the concretion. It is strongly concavo-convex, with the lateral edges ornamented with ovate ganoine tubercles and the midline ornamented with anteroposteriorly oriented ganoine ridges.

**Pectoral girdle.** The posttemporal is roughly triangular in shape, contacting the extrascapulars anteriorly. It is ornamented with ganoine tubercles. The presupracleithrum is a small element fitting between the posttemporal, dorsal opercle, extrascapular, and supracleithrum. Its surface is not well preserved. The supracleithrum is elongate and positioned at the very back of the skull. Preservation is quite poor. The postcleithral region is not well preserved, especially dorsally, but at least three postcleithra are present. These are ornamented with tubercles.

**Paired Fins.** Only the pectoral fin is preserved. It inserts low on the flank, slightly ventral to the opercle-subopercle contact, and consists of approximately 24 rays (Fig. 5E). These are for the most part unsegmented, and there is some evidence that at least the posterior rays bifurcated distally. The posterior rays are shorter than the anterior rays, as preserved. The leading edge of the first fin ray is lined with small fringing fulcra.

**Unpaired fins.** The only portion of the unpaired fins preserved are a few fragmentary proximal dorsal fin rays. These are positioned along the dorsal midline (assessed using scale morphology) with the most anterior preserved ray situated immediately posterior to vertical scale row 23.

**Squamation** (Fig. 4). Thirty-nine vertical scale rows are preserved along the lateral line, with an unknown number of vertical rows missing from the caudal peduncle. Anteriorly, each vertical row consists of at least 16 scales, and this increases to at least 24 scales at the level of the anterior part of the dorsal fin. The anterior dorsal scales have ganoine tubercles, especially concentrated along the anterior and dorsal borders of the scales. The scales close to the dorsal midline bear short ganoine rugae anteriorly, and more posterior scales bear large tubercles. Posterior to the opercle, only the first few scale rows show ganoine tubercles. The anteriormost ventral scales are heavily ornamented with ganoine tubercles, but more posteriorly these show a similar morphology to the scales posterior to the opercle. The scales posterior to the opercle are rectangular in outline when articulated, more than twice as high as long. Posterior to the first few rows, these are ornamented with a thin, smooth layer of ganoine punctuated by multiple pits (Fig. 4D), and along the anterior edge of the scale occasionally also more elongated pits. The ganoine layer becomes patchy in the posterior abdominal region (Fig. 4B). Dorsal to the lateral line, the scales are smaller and more equidimensional. Where disarticulated, it can be seen that the scales have a straight anterior edge ending in a dorsally directed process, and posterior to this also have a dorsal process (peg) which slots into a corresponding groove on the medial surface of the next scale in the vertical row (Fig. 4C). The lateral line canal is visible on the first scale posterior to the supracleithrum, but not on more posterior scales. This is partially, but not entirely, attributable to poor preservation of the scale surface. In the caudal peduncle, the scales become increasingly diamond-shaped, and the most posteriorly preserved scales are longer than wide.

**Occurrence.** The type and only known specimen was collected from the Pliensbach near Zell unter Aichelberg (48°38′30″N 9°35′15″E), District of Göppingen, Baden-Württemberg, Germany (Fig. 1), from the Opalinuston Formation, *Leioceras opalinum* Zone (Middle Jurassic, early Aalenian).

### Remarks

**Generic attribution.** *Dapedium* is notoriously difficult to diagnose, and of the characters outlined by Thies & Waschkewitz (2016), only the heavy cranial ornamentation of ganoine tubercles and ridges and 4–8 branchiostegal rays can be accurately assessed in SMNS 96990. However, *Dapedium ballei* is a deep-bodied fish distinguishable from the Toarcian species of *Dapedium* only in minor characteristics (see below) and lacking autapomorphies. As such, we are confident that it can be referred to this genus.

**Specific comparisons.** Thies & Waschkewitz (2016) compiled a table of characteristics diagnosing the Toarcian species of *Dapedium* from southwestern Germany. They considered axial fineness, width of the skull roof, fusion of elements in the orbital series, presence of a presupracleithrum, gular shape, opercle shape, presence of serrated scales, scale shape, and potentially number of lepidotrichia as diagnostic. Based on these characters, SMNS 96990 represents a species distinct from the named taxa from the Toarcian Posidonienschiefer Formation.

*Dapedium ballei* sp. nov. shares the sinusoidal shape of the anterior edge of the opercle with *D. caelatum*, but differs in that *D. caelatum* lacks the degree of size and shape differentiation between the abdominal scales above and below the lateral line seen in *D. ballei*, and has a shallower body profile, even given that only minimum body depth could be measured in *D. ballei*. *D. caelatum* also has slightly larger teeth (tooth height ∼5 mm, lower jaw length = 43 mm (SMNS 51906) vs. mandible length = 60 mm, tooth height = 4 mm in *D. ballei*). Thies & Waschkewitz (2016) noted the absence of a presupracleithrum as a diagnostic feature of *D. caelatum*, but this element is present in some specimens referred to this species (e.g., SMNS 51906), and its absence in the *D. caelatum* neotype may be due to taphonomic displacement (Thies, Hauff & Herzog, 2008).

*Dapedium stollorum* differs from *D. ballei* in having an opercle with a straight ventral contact with the subopercle and lacking the dorsoanterior projection. In addition, the trunk scales of *D. stollorum* have a serrated rather than a smooth posterior edge. We have also identified some additional characters with which to differentiate the taxa: *D. stollorum* has many more branchiostegal rays than *D. ballei* (six to eight vs. four), and the medial-most ray is not significantly differentiated from more posterolateral rays, unlike in *D. ballei* where the medial-most ray is large and plate-like. *D. stollorum* also has a substantially enlarged interopercle, lacking in *D. ballei*, and the nasal is less mediolaterally wide relative to anteroposterior length. The teeth are absolutely and relatively smaller in *D. ballei* (mandible length = 60 mm, tooth height = 4 mm) than those of large individuals of *D. stollorum* (tooth height in holotype ∼5 mm, lower jaw length = 36 mm: Thies & Hauff, 2011).

*Dapedium pholidotum* differs from *D. ballei* in having an opercle with a straight ventral contact with the subopercle, and lacking the dorsoanterior projection, and has much deeper, narrower scales dorsal to the lateral line in the abdominal region than those seen in *D. ballei*. In addition, *D. pholidotum* has slightly more numerous branchiostegal rays (six vs. four), and the medial-most ray is not differentiated from more posterior rays. The teeth of *D. ballei* are slightly smaller, proportionately, than those of *D. pholidotum* (tooth height 2–3 mm to a lower jaw length of 21–25 mm: Thies & Waschkewitz, 2016). The nasals in *D. pholidotum* also differ in shape from those of *D. ballei*, being much narrower medially than laterally. Differing from most other species of *Dapedium*, the posterior infraorbitals are very small or fused to suborbital bones in *D. pholidotum* and *D. ballei* (probably also in *D. colei* NHMUK PV P 4431; Wenz, 1968; Thies & Waschkewitz, 2016).

Two Toarcian species of *Dapedium* have also been named from France. *D. magnevillei* is poorly known but was described as having tuberculate ornamentation on all trunk scales (rather than punctate ornamentation, as in *D. ballei*), and also has a very high number of branchiostegal rays (seven vs. four) (Agassiz, 1833–1843). *D. milloti*, also from the Toarcian of France, is even more poorly described than *D. magnevillei*. The original description stated that the cranial elements and scales lacked visible ornamentation (Sauvage, 1891), however tuberculate cranial ornamentation was described for referred material (Wenz, 1967). This species requires redescription and revision to assess its validity relative to the Early Jurassic material from southwestern Germany before more detailed comparisons can be undertaken.

In comparison to the best-known Hettangian–Sinemurian species, *Dapedium ballei* differs from *D. politum* and *D. granulatum* in that in the latter species, the pectoral fin inserts ventral to the ventral edge of the interopercle, the maxilla is more robust, and the ornamented portion of the preopercle is much less than half the lateral exposure of the interopercle. In addition, in *D. politum* the posterior margin of the scales is serrated, and the ornamentation of the orbital and opercular series forms blotches rather than tubercles, whereas in *D. granulatum* the flank scales also bear tubercular ganoine ornamentation. *D. granulatum* has unusually small teeth relative to body size (Smithwick, 2015), whereas in *D. ballei* the teeth are somewhat larger (*D. granulatum*: mandible length = ∼73 mm, tooth height = 2 mm vs. *D. ballei* mandible length = 60 mm, tooth height = 4 mm). *D. radiatum* differs from *D. ballei* in having scales with finely pectinated caudal borders and the ornamentation of the skull bones consisting of ganoine tubercles and rugae, which are notably larger than in the new species.

Similarities of *Dapedium ballei* sp. nov. to *D. punctatum, D. angulifer,* and *D. colei* are much more pronounced than in the preceding taxa; the cranial ornamentation in particular is very similar. However, as in the previous species, the pectoral fins in *D. punctatum, D. angulifer,* and *D. colei* are situated in a more ventral position and the ornamented portion of the preopercle is much less than half the lateral exposure of the interopercle (Fig. 5). In addition, these taxa have only two large suborbitals contacting the opercle (rather than three). The posterior edges of the flank scales of *D. punctatum* are serrated, rather than smooth as in *D. ballei*.

Other Hettangian–Sinemurian species are poorly defined and very incompletely described. Among them, *Dapedium dorsalis* is described with almost smooth dermal bones, with only few small and sparse tubercles and reticulated markings on the opercle, but may be a juvenile (Woodward, 1895). The holotype of *D. orbis* (Agassiz, 1833–1843: Atlas, Tome II, Tab. 25d) cannot be located at present, but the expanded, anteroposteriorly compressed marginal teeth described by Agassiz do not closely match those of *D. ballei*.

The Middle Jurassic (Callovian)-aged *Heterostrophus phillipsi* has been suggested to be morphologically similar and closely related to *Dapedium* (Woodward, 1929; Gibson, 2016). However, *H. phillipsi* and *D. ballei* are clearly distinct, in that the subopercle in *D. ballei* is proportionately larger, the pectoral fin is positioned more ventrally, the anterior and ventral edges of the opercle are sinusoidal, and the branchiostegal rays are much more heavily ornamented with ganoine tubercles. The type and only other species of *Heterostrophus*, *H. latus* Wagner, 1863 from the Late Jurassic (Kimmeridgian–Tithonian) Solnhofen Archipelago also differs from *D. ballei* in the higher position of the pectoral fin and the generally much weaker ornamentation of the dermal bones, including only fine striations on the opercle and subopercle, and an almost smooth interopercle and branchiostegals. Furthermore, the scales of *H. latus* are finely ornamented with densely arranged delicate rugae, but there are no pits or tubercles as in *D. ballei*.

### Other specimens of *Dapedium* from the Opalinuston Formation

**Table:** 

**Figure 7:**
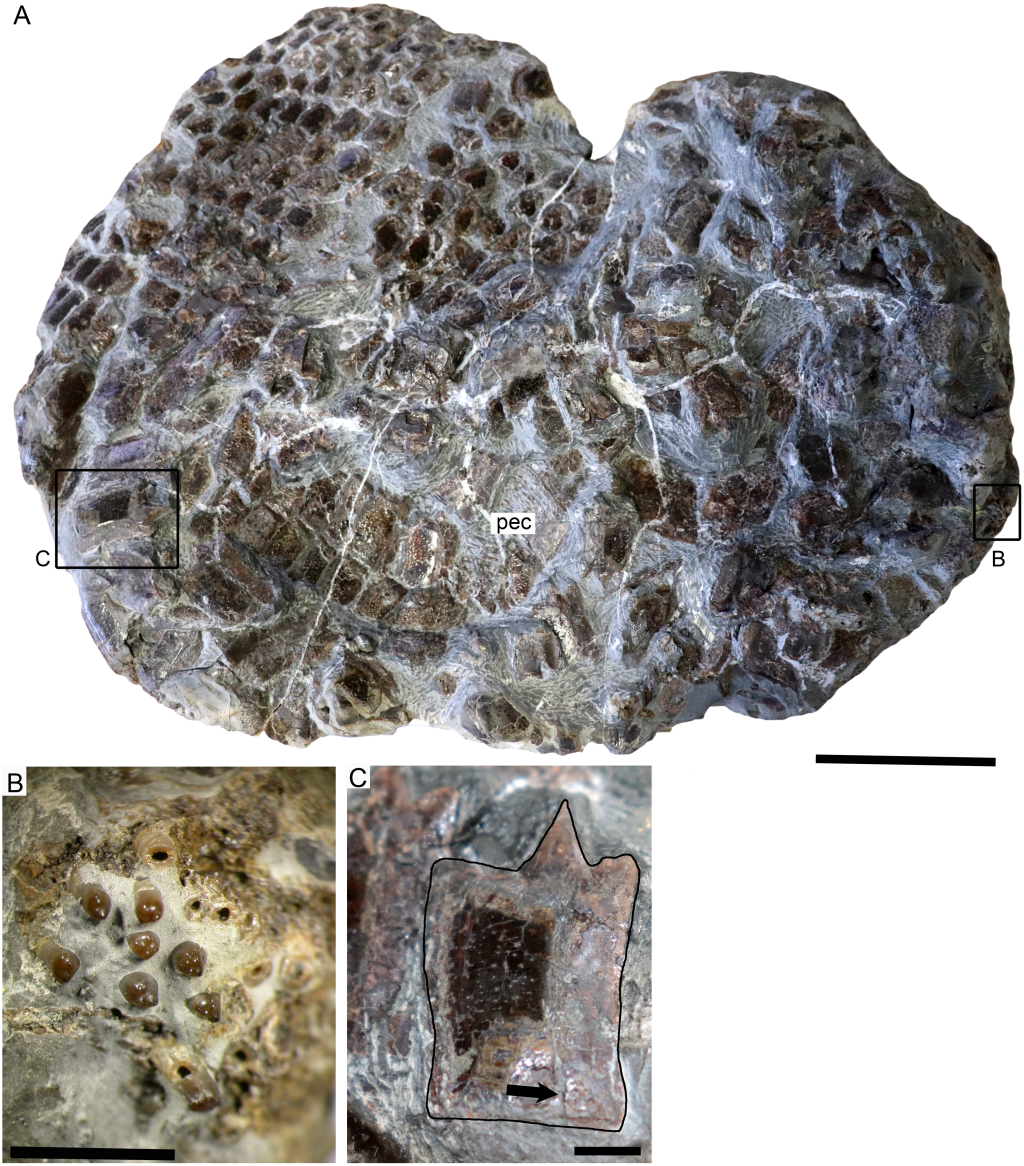
SMNS 50167, *Dapedium* sp. from the Opalinuston Formation of Baden-Württemberg. (A) overview image showing the location of insets (B) and (C). Anterior is to the right. (B) Palatal element bearing bi- to multicuspid teeth. (C) Dorsoventrally expanded flank scale showing punctate ganoine ornamentation (dark patch) becoming more irregular posteriorly. The ganoine has been eroded ventrally. The arrow indicates the groove for articulation with the preceding scale. Scale bar part (A) equals 50 mm, parts (B) and (C) equals 5 mm.

*Dapedium* sp.
Figure 7

Although very broken up, SMNS 50167 is presumed to have been a large individual as the measurements of the deep flank scales are similar to those of SMNS 96990. The skeleton has been prepared in right lateral view. Most of the flank scales remain in articulation, but the cranial elements are broken and disarticulated.

**Skull.** The cranial elements are badly jumbled and fragmented. Cranial elements are clearly ornamented with ganoine tubercles, but few bones can be identified due to breakage and overlap (Fig. 7A). A single dentigerous element is preserved; this is a tooth-bearing element from the palatal region characterised by stout, bi- to multicuspid teeth (Fig. 7B). The cusps are blunt and irregularly arranged, in an approximately linear arrangement on the tooth apex in some teeth and on others in a more molariform pattern. There is variation in cusp size and prominence within a tooth. Some of the cusps form centripetally oriented ridges in the centrally positioned tooth.

**Squamation.** The scales are identical in morphology to those of SMNS 96990, with more equidimensional scales above the lateral line and dorsoventrally deepened flank scales ventral to the lateral line in the dorsal region. Ganoine deposition is thin and uneven, and ornamentation is punctate. Isolated scales have a straight anterior edge ending in a dorsally directed process, and posterior to this also have a dorsal process (peg) which slots into a corresponding groove on the medial surface of the next scale in the vertical row (Fig. 7C).

**Paired fins.** A small portion of the right pectoral fin is preserved, adhering to the flank scales (Fig. 7A). This is interpreted as the distal portion of the pectoral fin, because all lepidotrichia are segmented into short blocks, something that does not occur in the proximal lepidotrichia in *Dapedium*. Small fringing fulcra are present along the leading edge. The lepidotrichia bifurcate at least once in the short section preserved.

**Remarks.** Based on stratigraphic occurrence, body size, and the morphology and ornamentation of the squamation, SMNS 50167 is consistent with *D. ballei* sp. nov. However, it is currently referred to *Dapedium* sp. due to the extremely fragmentary nature of the specimen.

**Table:** 

**Figure 8:**
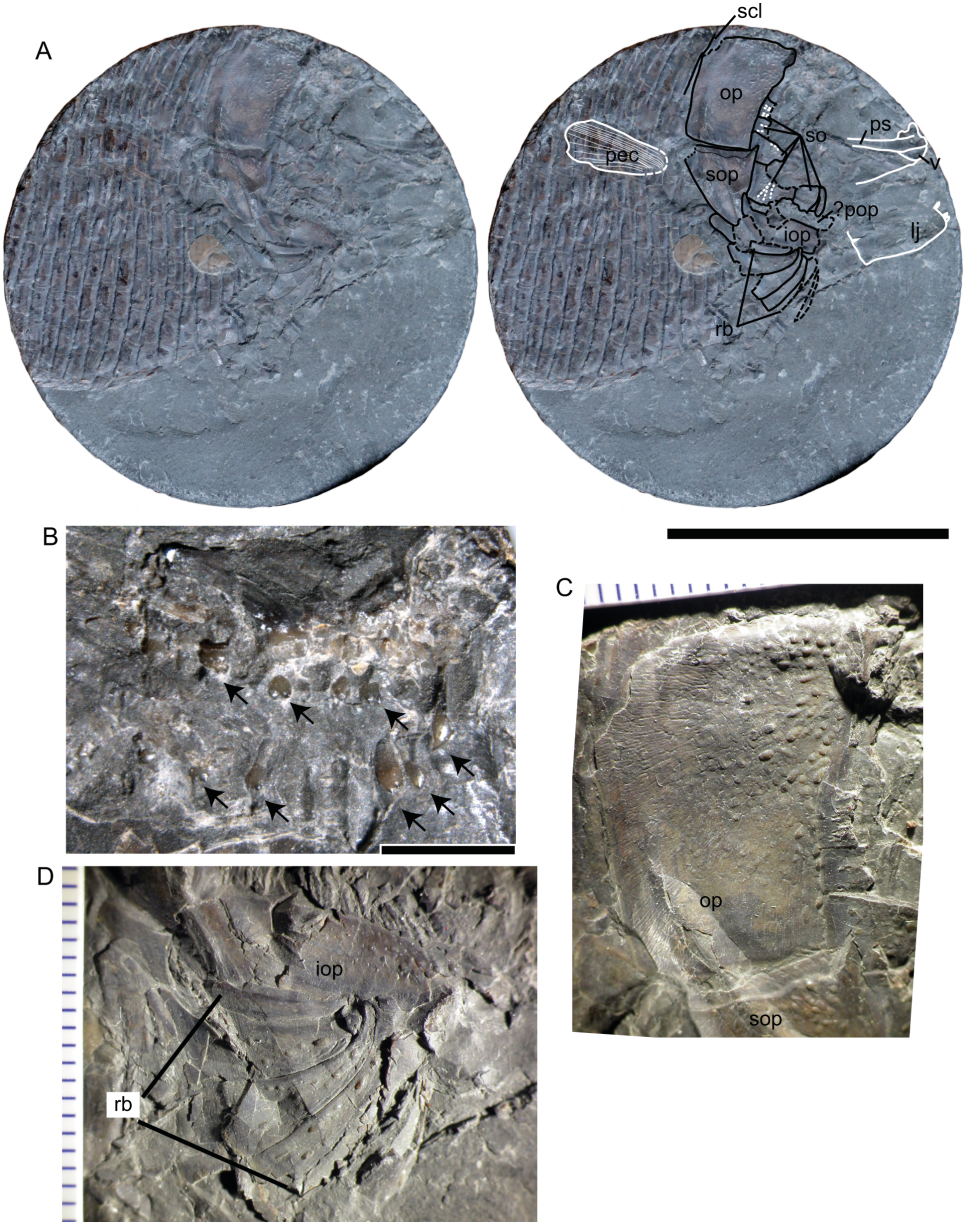
SMNS 13564, *Dapedium* sp. from the Opalinuston Formation of Baden-Württemberg. (A) photo and interpretation of the specimen. Structures indicated in white are preserved as impressions, those in black are preserved as bone. Large dashes indicate areas of breakage, small dashes indicate sensory canals. (B) palatal dentition illustrating multicuspid teeth; teeth are indicated with arrows. (C) Opercle, showing the developing ganoine ornamentation, with fully-formed tubercles restricted to the anterodorsal corner and polygonal structures more posteriorly interpreted as developing tubercles. (D) Branchiostegal rays, also showing weak ornamentation. Scale bars represent 50 mm (A), and 2 mm (B), respectively. *Abbreviations.* iop, interopercle; lj, lower jaw; op, opercle; pec, pectoral fin; pop, preopercle; ps, parasphenoid; rb, branchiostegal rays; scl, supracleithrum; so, suborbitals; sop, subopercle; v, vomer.

*Dapedium* sp.
Figure 8

SMNS 13564 is a very small individual (skull length 50.5 mm, estimated standard length = 157–178 mm). This specimen consists of the anteroventral portion of the fish only. The lower jaw is preserved as an impression in fine-grained sediment; the lateral portion of much of the skull, as well as the pectoral girdle and fin, are not preserved. The specimen appears to be exposed from the stratigraphically upper side, as indicated by the presence of encrusting organisms (a brachiopod, some very small diameter burrows, possibly serpulid).

**Suborbitals.** Evidence for a minimum of six suborbitals is preserved around the posterior orbital margin. Suborbitals are sparsely ornamented with ganoine tubercles. The largest element of the series is situated posteroventrally. This element carries radiating sensory canals oriented towards the orbit. These were most likely covered by a thin sheet of bone that collapsed during post-mortem compression. All more dorsally situated suborbitals also preserve evidence of sensory pits, either caved in or covered by a thin, translucent layer of bone.

**Palatal elements.** The parasphenoid is present as an impression on the sediment but provides no details. The vomers are preserved towards the anterior end of the block. Whether they are single or fused cannot be assessed. They are covered in relatively large, blunt, multicuspid teeth (Fig. 8B).

**Mandible.** The right mandible is preserved in medial view posteriorly and as an impression on the sediment anteriorly. A single broken styliform dentary tooth is present; additional teeth are preserved as impressions on the sediment.

**Preopercle, opercular bones, and branchiostegals.** The opercle is 21 mm deep and 14.6 mm long. It is ornamented with ganoine tubercles, most strongly near the anterodorsal articulation with the skull. Ganoine tubercles in the middle of the element have little relief, and tubercles are lacking towards the posterior edge (Fig. 8C). The opercle is roughly quadrangular in shape, with the anterior and ventral edges forming a 120° angle. The subopercle contacts the opercle along a more or less straight contact, with a small anterior dorsal process. On the anterior dorsal surface is a small region of tubercles, otherwise the subopercle is free from ornamentation. The preopercle is most likely absent. However, an area of broken bone between the ventral edges of the suborbitals and the interopercle may represent this element. The interopercle is exposed anterior to the subopercle. It is anamestic, with a few sparse ganoine tubercles on its anteriormost end. Otherwise it lacks ornamentation.

Five to six branchiostegal rays are present, the lateral four of which are well-preserved (Fig. 8D). These are ornamented with very few tubercles, except for the most posterior ray, which is unornamented. All rays are anteriorly slender and broaden posteriorly, and show strong lateral curvature.

**Pectoral fin.** The pectoral fin is preserved as an impression on the flank scales. Some distal lepidotrichial fragments remain; otherwise all skeletal structures associated with the pectoral fin appear to be with the missing counterpart. The pectoral fin appears to have inserted dorsal to the ventral edge of the interopercle, posterior to the subopercle.

**Squamation.** The flank scales are deeper than long, but are very thin such that they have been deformed around the leading edge of the proceeding scale. The posterior edge of the scales is smooth. Ganoine was apparently present, but ornamentation is difficult to determine with certainty. The ventral-most scales are dorsally deflected, forming a ventral keel. They are ornamented with lanceolate ganoine ridges.

**Remarks.** Little work has been undertaken on the ontogeny of *Dapedium*, despite the relatively rich fossil record for this genus. However, aside from relatively small body size, SMNS 13564 is characterised by numerous features interpreted as resulting from a young ontogenetic age, including visibility of the cranial sensory canal system and weak development of the dermal ornamentation.

The sensory canals in the suborbitals of SMNS 13564 are covered with very thin bone, so thin as to be translucent when viewed through the dissection scope, and the diameter of the canal is large relative to the thickness of the bone, causing it to collapse inward during compression. In extant non-teleostean actinopterygian fishes, the lateral line system of the skull is initially open in superficial grooves. Investiture of the lateral line system in the dermatocranium begins as ossification of an internal plate bearing a gutter-like groove, which eventually wraps around to enclose the canal (Jollie, 1984). In early ontogeny, the bone roofing the canal is initially thin, such that the canal is clearly visible beneath a thin, translucent layer, but in later stages is thickened, such that the canal is not visible in external view, and is proportionately narrower relative to the size of the element (compare e.g., Grande & Bemis, 1998: figs. 17 and 20).

The dermal skull bones of SMNS 13564 are predominantly smooth, ornamented with sparse ganoine tubercles. This is very different from the dermal ornamentation described for both *D. ballei* and Toarcian *Dapedium* species. A simple increase in the number and density of ganoine tubercles on the cranial bones with increasing body size has been documented for other species of *Dapedium* (e.g., *D. politum*: Woodward, 1895).

In addition to dermal ornamentation, SMNS 13564 differs from *Dapedium ballei* sp. nov. in several details. These include the shape of the anterior and ventral edges of the opercle, and the number and relative size of the branchiostegal rays. Opercular shape in particular has been used to differentiate Early Jurassic *Dapedium* species (Thies & Waschkewitz, 2016); however, the influence of ontogeny on this feature has not been investigated in *Dapedium*. Multiple sympatric species of *Dapedium* are present in all Early Jurassic localities from which the genus is adequately known (Forey, Longbottom & Mulley, 2010; Thies & Waschkewitz, 2016), and this possibility cannot be ruled out for the Opalinuston Formation. For that reason, we consider SMNS 13564 to be referable only to *Dapedium* sp.

## Discussion

### Heterostrophus

*Heterostrophus* was described based on a fusiform fish from the Late Jurassic Solnhofen Archipelago of Bavaria, Germany (*H. latus* Wagner, 1863). A second species (*H. phillipsi*) was later named based on material from the Middle Jurassic of England (Woodward, 1929). Similarities to *Dapedium* have been noted, especially with regard to *H. phillipsi*, and include overall similarities in body shape and cranial morphology, tubercular cranial ornamentation and the shape and ornamentation of the body scales, as well as the presence of a presupracleithrum (element *x* of Woodward, 1929). However, generic distinctness was maintained based on several features. These include (1) increase in the number of elements in the suborbital series (discussed in more detail below), (2) decreased ganoine thickness on the trunk scales, (3) pectoral fins placed higher on the flank (discussed in more detail below), and (4) reduced pelvic fins (Woodward, 1929). *Heterostrophus phillipsi* falls outside of the range of variation seen in *Dapedium* spp. in all of these features*. Heterostrophus latus* is further removed morphologically, sharing with *H. phillipsi* the high position of the pectoral fins, but differing from it and from the species of *Dapedium* in the relative size of the subopercle, which is almost as deep as the opercle in *H. latus*, the general shape of the lower jaw with a comparatively much lower symphysis, and the weak ornamentation of the opercular and branchiostegal bones and fine ornamentation of the scales.

### New morphological information for *Dapedium*

The study of *Dapedium ballei* led us to the recognition of some anatomical features that were previously unknown in *Dapedium*.

**Articular process of the maxilla.** In the strongly ossified skull of *Dapedium*, the maxilla is normally well articulated and only the lateral surface of the bone is exposed (Fig. 6A). This lateral surface is heavily ornamented and produces a stout anterior end, which is attached to the premaxilla when the mouth is closed, thus giving the impression, as suggested by Wenz (1967), that these bones are sutured and there is no anterior articular process. The maxilla is disarticulated in SMNS 96990 and the well-developed, anteromedially directed articular process is fully exposed (Fig. 6D). We have also found the maxillary articular process in *D. pholidotum* (SMNS 87405, 87415: Figs. 6B–6C) and *D. caelatum* (SMNS 56226).

**Posterovertical process of the antorbital.** *Dapedium ballei* sp. nov. shows an antorbital bone with a posterovertical process. The antorbital is relatively poorly preserved, but the complete bone with the process including a sensory canal is well visible under UV-lighting (E Maxwell, pers. obs., 2017). Specimen SMNS 53978 of *D. pholidotum* also exhibits a very well preserved antorbital with a posterovertical process including a sensory canal (Fig. 6A). Therefore, although such a posterovertical process has not been described previously for any species of *Dapedium*, it might be present in other species of the genus.

### Evolutionary trends in *Dapedium*

As a genus, *Dapedium* is relatively conservative, showing only minor changes over its ∼35 million year history. Here, we describe some trends seen in the genus, and also mention comparisons with *Heterostrophus,* where appropriate. The phylogenetic meaning of these morphological changes should be explored in the framework of a cladistic analyses, which is beyond the scope of the present contribution. No comprehensive species-level phylogenetic hypothesis is available for *Dapedium*.

**Expanded external exposure of the preopercle** (Fig. 5). This characteristic is particularly noteworthy, as it is one of the only features showing a consistent trend-like trajectory through time in *Dapedium*, in spite of some minor intraspecific variation. In *Dapedium noricum*, the preopercle lacks any external exposure, being entirely covered by the suborbital series (Tintori, 1983). In *D. colei*, external exposure of the preopercle is extremely minimal (Smithwick, 2015), and is marginally expanded in *D. granulatum* and *D. punctatum* (Fig. 5A). Exposure is consistently greater in the Toarcian species (*D. caelatum*, *D. stollorum,* and *D. pholidotum* (Thies & Hauff, 2011; Thies & Waschkewitz, 2016); Figs. 5B–5D), only slightly greater than in the Sinemurian-aged *D. politum* (Smithwick, 2015). Preopercle exposure in *D. ballei* (Fig. 5E) is similar to Toarcian *Dapedium* species. In *Heterostrophus phillipsi,* preopercle exposure is greatly expanded relative to *Dapedium* (Fig. 5F). A reduction in the exposure of the interopercle is directly related to the increase in the exposure of the preopercle.

**Fragmentation of the suborbital series.** Variation within species in the number of elements in the suborbital series is relatively high in *Dapedium*, but shows a weak increase through time between species. *D. noricum* was described as having four to five elements in the suborbital series (Tintori, 1983). Among some Hettangian–Sinemurian forms, this increases to six (*D. punctatum*, *D. politum*), seven (*D. colei*), or eight (*D. granulatum*) (Forey, Longbottom & Mulley, 2010). Seven suborbitals are present in *D. caelatum* (E Maxwell, pers. obs., 2017), while nine have been described for *D. pholidotum* (Thies & Waschkewitz, 2016), and eight are observed in *D. ballei*. The greatest variation is observed in *D. stollorum*, in which six to 10 suborbitals have been documented. *Heterostrophus phillipsi* appears to have 12 or more elements in the suborbital series (based on Woodward, 1929: pl. 1). In gars, the number of suborbital bones increases both over ontogeny in *Lepisosteus* and *Atractosteus*, and over phylogeny (Grande, 2010: page 758, table 64). This is in reverse to the trend observed in the Triassic ginglymodian *Ticinolepis longaeva*, in which variation in the number of suborbitals also occurs intraspecifically, but with a decrease in number through time (López-Arbarello et al., 2016).

**Position of the pectoral fins.** There is evident variation in the relative position of the pectoral fins among the species of *Dapedium*. Ideally, the relative position of these fins should be represented with relative morphometric measurements, but this is problematic due to incomplete preservation of many specimens. The pectoral girdle is attached to the skull dorsally and, as a whole, the dermal components have a stable position relative to the skull, with the anteromedial portion of the cleithrum serving for attachment of the sternohyoideus muscle. Among the skull bones, although the whole operculo-gular series extends from the anterodorsal articulation of the opercle with the hyomandibula to the attachment of the most anterior branchiostegal onto the ventrolateral border of the anterior ceratohyal, the relative size of the opercle, subopercle and branchiostegals is highly variable among the species of *Dapedium* and *Heterostrophus*. The interopercle, however, has a stable position between the branchial cover and the lower jaw among dapediids, thus serving as a valid reference for the relative position of the pectoral fin. In *Dapedium noricum*, the pectoral fins insert low on the flank, below the ventral edge of the interopercle and the caudal peduncle, and are oriented horizontally (Tintori, 1983). In the Hettangian–Sinemurian species, this position of the fins remains similar to that in *D. noricum* (Fig. 5A). Among the Toarcian species, *D. stollorum* most resembles the Hettangian–Sinemurian species in fin position (Fig. 5B). The pectoral fins of *D. pholidotum* have shifted dorsally relative to *D. stollorum* and geologically older species, and insert dorsal to the ventral edge of the interopercle, posterior to the ventral third of the subopercle (Fig. 5C; Thies & Waschkewitz, 2016), and in *D. caelatum* the pectoral fin inserts dorsal to the ventral edge of the interopercle, slightly ventral to the opercle-subopercle contact (Fig. 5D), and also dorsal to the ventral edge of the caudal peduncle. In *D. ballei*, the position of the pectoral fin is similar to that of *D. caelatum* (Fig. 5E). The pectoral fin of *Heterostrophus phillipsi* has been further dorsally shifted and inserts well above the level of the ventral border of the interopercle (Fig. 5F; Woodward, 1929).

**Increase in the number of scale rows along the horizontal midline.** This character is more variable within species and variation between species through time is less remarkable than that of the external exposure of the preopercle, but there does appear to be a slight increase in the number of scale rows over time in *Dapedium*. Forty scale rows are documented in *D. noricum*, 40–44 in *D. politum*, 43 in *D. angulifer*, 44 in *D. colei*, and 47 in *D. granulatum* (Deecke, 1888; Tintori, 1983; Forey, Longbottom & Mulley, 2010). In the Toarcian species, these numbers increase to 46–48 in *D. pholidotum*, 48 in *D. caelatum*, and 53 in *D. stollorum* (Thies & Waschkewitz, 2016). This may correlate with increasing vertebral counts in the genus.

**Body size.** *Dapedium* has been described as a small- to mid-sized fish up to 45 cm in standard length (Thies & Hauff, 2011), and as Table 1 suggests the size range observed in the genus is highly variable. The smallest species is *D. noricum*. The largest species is *D. granulatum*, and *D. granulatum* is the only Early Jurassic species to attain a standard length of greater than 450 mm (standard length for *D. angulifer* was estimated at ∼425 mm from a high-resolution photograph). Therefore *D. ballei* is one of the largest species in the genus. It should be noted that although differences in standard length between *Dapedium* species may be relatively slight, differences in mass are proportionately greater due to the deep-bodied outline of these fishes.

### Palaeoecology

*Dapedium* has been suggested to be a facultatively durophagous generalist feeder (Thies & Hauff, 2011; Smithwick, 2015). Although dramatic differences in tooth size between species have previously been noted (Smithwick, 2015) these were not speculated to be related to differences in diet or feeding behaviour between species. Thus, the relatively small tooth size differences observed between *D. ballei* and the Toarcian *Dapedium* species are unlikely to relate to significant dietary changes. Contrasting interpretations also exist as to where in the water column *Dapedium* lived, with some authors favouring a bottom-feeding interpretation (Aldinger, 1965) while others hypothesise that *Dapedium* lived high in the water column, based on environmental factors (Böttcher, 1998) and the absence of anterior procumbent prehensile teeth in the genus (Tintori & Lombardo, 2018). The similarly-shaped dapediid *Hemicalypterus* had higher, more procumbent spatulate teeth that are hypothesized to have functioned in benthic feeding (Gibson, 2016). The pectoral fin in this genus is positioned even lower on the flank than in *D. noricum*, although in the case of *Hemicalypterus* this may be a primitive characteristic.

The correlation between pectoral fin position and habitat use is complex, and has not been comprehensively investigated among extant species. Among deep-bodied fishes, placement of the pectoral fin closer to the lateral midline has been associated with a reduction in the pelvic fins and a steady-swimming locomotor strategy (as found in pelagic fishes), whereas lower placement of the pectoral fins and enlarged pelvic fins has been suggested to be more typical of reef fishes (Breder Jr, 1926). A morphometric study of sparids suggests that species consuming a mixture of benthic and pelagic prey had a pectoral fin positioned closer to the lateral midline than reef species and those consuming primarily benthic prey (Antonucci et al., 2009: figs. 5–6).

The observed dorsal shift in pectoral fin position through time in *Dapedium* and *Heterostrophus* has been associated with the shift from carbonate platform to more pelagic habitats (Woodward, 1929; Tintori & Lombardo, 2018). Fluctuating oxygen levels in the lower water column during the deposition of the Opalinuston Formation make both a necktobenthic and pelagic interpretation equally possible for *D. ballei* (Hegele, 1995); however the relatively small premaxillary teeth and dorsally displaced pectoral fin argue against a demersal or necktobenthic habit for the species.

Overall, the vertebrate fauna of the earliest Middle Jurassic of the Southwest German Basin bears strong similarities to the Early Jurassic Posidonia Shale fauna, with most observed differences noted primarily at the species level (e.g., a hybodontid chondrichthyan; Quenstedt, 1856–1858), the actinopterygians *Saurorhynchus* (Maxwell, 2016), and a lepidotid (anecdotal report: Hegele, 1995), the early teleostean *Leptolepis* (otoliths only: Schwarzhans, 2018) as well as at least one species of *Dapedium* (current contribution), the ichthyosaur *Stenopterygius* (Maxwell, Fernández & Schoch, 2012) and an undescribed temnodontosaurid ichthyosaur, the thalattosuchian *Steneosaurus* (anecdotal report: Hegele, 1995), and indeterminate sauropterygian remains (reviewed by Vincent, Bardet & Morel, 2007). However, some changes in the ecological composition of the chondrichthyan fauna have been noted (Kriwet, 2003), and it is possible that further diagnostic finds will decrease perceived similarities between the faunas.

## Conclusions

*Dapedium ballei* represents the geologically youngest find of the genus *Dapedium*, extending the stratigraphic range of the genus from the early Toarcian to the early Aalenian (almost 7 Myr) and increasing the number of valid species in the genus to 15. *D. ballei* is also one of the largest species in the genus, at ∼50 cm in standard length. However, *Dapedium* is a morphologically and ecologically conservative genus, making the identification of diagnostic characters challenging. In spite of its long (∼35 million year) stratigraphic range, *Dapedium* was apparently a poor disperser, and has not been reported outside of Europe. The Opalinuston Formation fills an important gap in the marine vertebrate fossil record between the well-described Posidonia Shale and Oxford Clay lagerstätte, and finds from this horizon have the potential to greatly improve our understanding of evolutionary dynamics over this period of faunal transition.

## Institutional abbreviations

BGS: British Geological Survey, London, UK
NHMUK: Natural History Museum, London, UK
SMNS: Staatliches Museum für Naturkunde Stuttgart, Germany
SNSB-BSPG: Staatssammlung für Paläontologie und Geologie, München, Germany
WARMS: Warwickshire Museum, UK

## Additional Information and Declarations

### Data Availability

The following information was supplied regarding data availability: The fossil materials described in the manuscript are accessioned and stored in the collections of the Staatliches Museum für Naturkunde Stuttgart, Rosenstein 1, 70191 Stuttgart, Germany.

### New Species Registration

The following information was supplied regarding the registration of a newly described species: Species name: urn:lsid:zoobank.org:act:9ADC2848-FD80-4308-AAFE-294C09B0D28D Publication LSID: urn:lsid:zoobank.org:pub:2D6609C8-7EFD-4CBD-8ADF-49DB9DA65D97
